# Targeted Metabolomic and Transcript Level Analysis Reveals Quality Characteristic of Chinese Wild Grapes (*Vitis davidii* Foex)

**DOI:** 10.3390/foods9101387

**Published:** 2020-10-01

**Authors:** Yan-lun Ju, Xiao-feng Yue, Xue-ying Cao, Yu-lin Fang

**Affiliations:** 1 College of Enology, Northwest A & F University, Yangling 712100, China; juyanlun2016@nwsuaf.edu.cn (Y.-l.J.); yuexiaofeng@nwsuaf.edu.cn (X.-f.Y.); cxy13023330303@163.com (X.-y.C.); 2 Shaanxi Engineering Research Center for Viti-Viniculture, Yangling 712100, China; 3 Heyang Viti-viniculture Station, Northwest A & F University, Yangling 712100, China

**Keywords:** spine grape, targeted metabolomics, glycosidically bound volatiles, anthocyanins, gene expressions

## Abstract

Native to China, spine grapes (*Vitis davidii* Foex) are an important wild grape species. Here, the quality characteristics of one white and three red spine grape clones were evaluated via targeted metabolomic and transcription level analysis. Xiangzhenzhu (XZZ) had the highest soluble sugar and organic acid content. Malvidin-3-acetyl-glucoside and cyanidin-3-glucoside were the characteristic anthocyanins in spine grapes, and significant differences in anthocyanin composition between different clones were detected. Anthocyanins were not detected in Baiyu (BY) grapes. The transcript levels of *VdGST*, *VdF3′H*, *VdOMT*, *VdLDOX*, and *VdUFGT* were significantly related to the anthocyanin biosynthesis and proportions. A total of 27 kinds of glycosidically bound volatiles (including alcohols, monoterpenes, esters, aldehydes, ketones, and phenolic acid) were identified in spine grapes, with Gaoshan #4 (G4) and BY grapes having the highest concentrations. The *VdGT* expression levels were closely related to glycosidically bound volatile concentrations. These results increase our understanding of the quality of wild spine grapes and further promote the development and use of wild grape resources.

## 1. Introduction

Spine grapes (*Vitis davidii* Foex) are native to China and belong to the East Asian *Vitis* spp. They are so named because the canes are densely covered with 1–2 mm thorns [1]. Spine grapes are mainly found in the Yangtze River Basin and Yunnan–Guizhou Plateau in South China, including Yunnan, Jiangxi, Hunan, and Chongqing Provinces [2,3]. In recent years, researchers have discovered many kinds of wild spine grapes, mainly red grapes, as well as a few white mutants. The researchers named them “Xiangzhenzhu”, “Gaoshan”, and “Baiyu” [4]. Spine grapes are rich in phenolics and have strong antioxidant power [5,6]. As a good source of phenolics and free aromatics, spine grapes have been cultivated widely in South China. Unlike *V. vinifera* grapes, spine grapes are mostly consumed as fresh fruits, so there are good commercial prospects for the development and use of spine grapes. More study on the characteristics of spine grapes is needed to provide a theoretical basis for their commercial use.

Previous studies on spine grapes have mainly focused on their phenolic content, antioxidant activity, and free volatiles [5]. In contrast, the most important quality metabolites in grape berries—soluble sugars, organic acids, anthocyanins, and glycosidically bound volatiles (GBVs)—have rarely been mentioned. Qualitative and quantitative knowledge of soluble sugars, organic acids, anthocyanins, and GBVs in spine grapes is limited and incomplete. Meng et al. [6] investigated the total phenolic content and antioxidant activity of four spine grape cultivars in Chongyi County (China), while Meng et al. [7] and Ju et al. [8] detected free volatiles in wild spine grapes. However, these studies only reported the phenolics or free volatile profiles of wild spine grapes, while other metabolites—especially soluble sugars, organic acids, and GBVs—were not involved, although these metabolites play an important role in grape and wine quality [9,10,11]. The anthocyanins in grape skins can be transferred into wine by macerating during fermentation and are the main source of color in wines. The anthocyanins, which can be combined with tannins in wine, play important roles in the stability of wine color, which give the wine a good taste and color, attracting consumers [12]. On the other hand, the aroma substances of wine mainly come from grape berries, so the contents and types of aroma substances in grape berries determine the quality and type of wine aroma [7,11] Many grape cultivars mature at different time periods. Their soluble sugar, organic acid, anthocyanin, and GBV profiles are assumed to differ based on the differences in varieties and growth environment [13,14,15]. It would be beneficial for the development and use of wild grape resources if the varietal metabolite differences were clarified.

Four representative spine grapes clones in South China were therefore identified in the present study for analysis of their (1) soluble sugar content, organic acid content, and the expression levels of sugar unloading-associated genes, such as *VdHTs*, *VdcwINV*, *VdGIN1*, *VdGIN2*; (2) anthocyanin profiles and the expressions of key genes, such as *VvPAL*, *VvC4H*, *VvCHS*, *VvF3′H*, *VvF3′5′H* etc., involved in anthocyanin biosynthesis; (3) the composition and content of GBVs and the expression levels of *VdGT5*, *VdGT6*, *VdGT7*, *VdGT9* and *VdPNGer*, which are associated with glycosidically bound volatiles biosynthesis; (4) the correlation among metabolites and gene expression levels.

## 2. Materials and Methods

### 2.1. Grape Samples

Four samples of *Vitis davidii* Foex, including three red clones—Gaoshan #2 (G2), Gaoshan #4 (G4), and Xiangzhenzhu (XZZ)—and one white clone, Baiyu (BY), were collected at commercial maturity from a commercial vineyard (Hunan province, South China, 109°32′ E, 27°29′ N). For each sample, collection was carried out by picking small groups of 4–6 grape berries from different parts of each cluster, for a total mass of about 1000 berries. All samples were frozen in liquid nitrogen immediately and stored at −80 °C before analysis.

### 2.2. Chemicals and Standards

All of the standards for soluble sugars, organic acids, anthocyanins, and GBVs were purchased from Sigma-Aldrich (Shanghai, China). The purity of these standards was ≥97%. Formic acid and acetonitrile (HPLC grade) were purchased from Fisher Co. (Fairlawn, NJ, USA). All other chemicals used were analytical grade or above and were purchased from Tianjin Kermel Chemical Reagent Co., Ltd. (Tianjin, China).

### 2.3. Analysis of Spine Grape Physicochemical Parameters

A digital Atago PAL-1 m (Atago Co. Ltd., Tokyo, Japan) was used to analyze the total soluble sugar (°Brix) content of the samples. Titratable acid was determined according to the methods of Wang et al. [16]. The pH value was determined by a Mettler Toledo FE20 Desktop pH meter (Mettler Toledo Instruments Co. Ltd., Shanghai, China). The determination of total anthocyanins was performed on the basis of the pH differential methods of Ju et al. [17]. Three technical replicates were analyzed for each sample.

### 2.4. HPLC Analysis of Spine Grape Soluble Sugars and Organic Acids Profiles

About 100 berries from each sample were deseeded, and the juice was squeezed out of them; 10 mL of juice was then centrifuged at 8000 rpm, 4 °C for 10 min. After centrifugation, 5 mL supernatant was filtered through a 0.45 μm inorganic filter and directly subjected to high-performance liquid chromatography (HPLC; LC-30A, Shimadzu Co., Ltd., Kyoto, Japan) for further analysis. A differential refraction detector (RID) and ZORBAXSD-C_18_ column (Agilent, 4.6 mm × 250 mm, 5 μm, Santa Clara, CA, USA) were used to determine soluble sugars. Injection volume was 10 μL. The column temperature was 40 °C. The phase was acetonitrile/water (*v/v* 80/20), the flow rate was 1.0 mL/min, and it was analyzed for 20 min. To determine organic acid content, a diode array detector (DAD) and Thermo Hypersil COLD aQ column (4.6 mm × 250 mm, 5 μm, Waltham, MA, USA) were used. Injection volume was 20 μL. The column temperature was 25 °C. The phase was 0.01 mol/L KH_2_PO_4_ (pH:2.55)/methanol (*v/v* 97/3), the flow rate was 0.5 mL/min, and it was analyzed for 20 min. Identification and quantification of soluble sugars and organic acids were according to the calibration curve of their standards. The concentrations of the stock solutions of fructose (*y* = 114,613*x* + 30,526, *R*^2^ = 0.9959) and glucose (*y* = 152,694*x* + 33,399, *R*^2^ = 0.9967) were 100 g/L, and the concentrations of the working solutions of fructose and glucose were 1 g/L, 2.5 g/L, 5 g/L, 10 g/L, 25 g/L, 50 g/L and 100 g/L, respectively. The linear ranges of fructose and glucose were from 2.5 g/L to 100 g/L. The concentrations of the stock solutions of tartaric acid (*y* = 4 × 10^6^*x* + 1 × 10^6^, *R*^2^ = 0.9971) and malic acid (*y* = 2 × 10^6^*x* + 1 × 10^6^, *R*^2^ = 0.9961) were 100 g/L, and the concentrations of the working solutions of tartaric acid and malic acid were 0.5 g/L, 1 g/L, 2.5 g/L, 5 g/L, 10 g/L and 20 g/L, respectively. The linear ranges of tartaric acid and malic acid were from 1 g/L to 10 g/L. The concentration of the stock solution of citric acid (*y* = 3 × 10^6^*x* + 153,828, *R*^2^ = 0.9999) was 1 g/L, and the concentrations of the working solutions were 0.03 g/L, 0.06 g/L, 0.12 g/L, 0.24 g/L, 0.48 g/L and 0.98 g/L, respectively. The linear range was from 0.03 g/L to 0.48 g/L. Three technical replicates of each sample were analyzed.

### 2.5. HPLC–DAD Analysis of Spine Grape Anthocyanin Profiles

The extraction of anthocyanins from spine grapes was completed according to our previous methods [18], the berry skins removed from fifty grape berries were freeze-dried at t 50 °C for the extraction of anthocyanins. Anthocyanin profiles were determined according to the methods of Bindon et al. [19] and Martín-Gómez et al. [20]. Briefly, an HPLC–DAD (Shimadzu Co., Ltd., Kyoto, Japan) system fitted with a C_18_ column (4.6 mm × 250 mm, Shimadzu Co., Ltd., Kyoto, Japan) was used to analyze the anthocyanin profiles. The conditions were as follows: mobile phase A: formic acid: acetonitrile: water (*v/v/v*) = 7:10:83; mobile phase B: formic acid: acetonitrile: water (*v/v/v*) = 2:54:44. The elution procedure was as follows: 0–15 min, 0–30% B; 15–25 min, 30–50% B; 25–35 min, 50% B. The flow rate of the mobile phase was 1.0 mL/min; the column temperature was 30 °C; the detection wavelength was 525 nm; the wavelength was scanned at 200–600 nm; the injection volume was 20 μL. Malvidin-3-glucoside (*y* = 0.00002*x* + 0.3689, *R*^2^ = 0.9999) was used as the internal standard to quantify the anthocyanins. The concentration of the stock solutions of standard was 400 mg/L, and the concentrations of the working solution were 0.054 mg/L, 0.13 mg/L, 0.27 mg/L, 0.54 mg/L, 0.9 mg/L, 1.5 mg/L, 2.5 mg/L, 5 mg/L, 10 mg/L, 25 mg/L, 50 mg/L, 100 mg/L and 400 mg/L, respectively. The linear range was from 0.27 mg/L to 400 mg/L.

### 2.6. GC–MS Analysis of Spine Grape Glycosidically Bound Volatiles

GBVs were extracted according to the methods of Wen et al. [21]. Briefly, 3 mL supernatant obtained above was added to a Cleanert PEP-SPE column (200 mg/6 mL, Agela, Wilmington, DE, USA), which was activated with methanol (10 mL) and distilled water (10 mL) before use. The soluble sugars and organic acids were eluted by 5 mL distilled water, and then 5 mL dichloromethane was added to wash out the free aroma. Finally, 20 mL methanol was added to elute the GBVs at a flow rate of 2 mL/min, which was collected in a 50 mL round bottom flask. To obtain the GBVs, the solvent in the flask was removed under vacuum at 30 °C, then 5 mL citric acid solution (pH:5) and 100 μL AR2000 (Rapidase, Seclin, France) were added separately. The mixtures were incubated for 16 h at 40 °C before analysis.

A Thermo-Finnigan TRACE1310-ISQLT GC–MS instrument (Thermo Finnigan, Bremen, Germany) was employed to analyze the GBVs. The TraceGOLD TG-5MS column (60 m × 0.25 mm I.D., 0.25 μm, Thermo Finnigan, Bremen, Germany) was used. The GBVs were extracted using headspace solid-phase micro-extraction fiber (HS-SPME, DVB/CAR/PDMS 2CM, 50/30 μm) in the headspace of the vials at 40 °C for 30 min. The fiber was desorbed for 4 min at 250 °C. The oven temperature was 50 °C (for 1 min), then increased to 130 °C at 3 °C/min and held there for 5 min, and then increased to 220 °C at 3 °C/min and held for 5 min. The transfer line temperature was 250 °C, and the ion source temperature was 280 °C. The mass spectrometer was scanned from 20 m/z to 450 m/z with an electron impact (EI) mode at 70 eV.

GBV identification was according to the NIST02 library and retention indices of the authentic standards. The internal standard was 2-octanol. The concentration of the stock solution of 2-octanol was 1 g/L, and the concentration of the working solution was 4 μg/L. The GBV calibration curves were built using the area ratio of target compounds to the internal standard against the concentration ratio. GBV quantification was carried out using these calibration curves.

### 2.7. Gene Expression Analysis by qRT-PCR

The relative expressions of the genes involved in the pathways for sugar unloading, anthocyanin biosynthesis, and GBV biosynthesis were determined by qRT-PCR. Total spine grape RNA was extracted using the extraction kit (Bioteke #RP3302, Beijing, China) [22]. First-strand cDNA was reverse-transcribed by Hiscript II Q RT SuperMix for qRT-PCR (Vazyme #R223-01). The qRT-PCR reaction was performed using an iQ^TM^5 Connect Real-Time System (Bio-Rad, Hercules, CA, USA). The specific primers used in this study are shown in Table S1 (Supplementary Materials), and *VdGAPDH* was used as the internal reference gene to analyze the gene expression levels. The 2^−ΔΔCT^ method was used to analyze the qRT-PCR data. All samples were analyzed in triplicate.

### 2.8. Statistical Analysis

All data are shown as mean ± SD. One-way ANOVA was performed to assess differences among samples using Duncan’s multiple range test with a significance level of *p* < 0.05. Data were analyzed using Excel and SPSS 21.0 software (SPSS Inc., Chicago, IL, USA). Multivariate statistical analysis was performed by MetaboAnalyst 3.0 (http://www.metaboanalyst.ca/) [23].

## 3. Results and Discussion

### 3.1. Physicochemical Parameters of Spine Grapes

To examine the differences between spine grape clones, physicochemical parameters—including soluble solids content (°Brix), pH, titratable acid, and total anthocyanin content—were analyzed. As Figure S1 (Supplementary Materials) shows, the soluble solids content of spine grapes ranged from 12.2 g/L to 15.4 g/L, which was lower than *Vitis vinifera* cultivars such as Cabernet Sauvignon (about 21 g/L–27 g/L in China), Merlot (about 21 g/L–25 g/L in China), and Chardonnay (about 20 g/L–25 g/L in China). This was consistent with the report by Meng et al. [7], which reported that the soluble solids content of four spine grape varieties ranged from 14.7 g/L to 16.0 g/L. The titratable acid content ranged from 2.26 g/L to 3.46 g/L. G4 grapes had the highest titratable acid content, while BY grapes had the lowest. The pH values were not always consistent with the titratable acid content. For example, the pH value was 3.86 for XZZ grapes with titratable acid at 3.11 g/L, while the pH value was 3.80 in BY grapes with titratable acid at 2.26 g/L. Yang et al. [24] also found that pH values were not consistent with the titratable acid content in *Vitis vinifera* cultivars such as Cabernet Sauvignon. The pH value of grape must does not change significantly during the fermentation process, so the pH of the grape berries determines the pH of the wine. In general, the pH value of *V. vinifera* wine ranges from 2.8 to 3.8, and appropriate pH value is important for the stability of the color of the wine [14]. Anthocyanins are crucial quality factors for grapes and wine [17]. In the present research, the higher total anthocyanin contents were found in spine grapes (20 g/kg–33 g/kg) than Cabernet Sauvignon grapes (12 g/kg–16 g/kg) [17,25]. XZZ grapes had the highest total anthocyanin content, while anthocyanins were not detected in BY grapes. The higher total anthocyanin content in spine grapes might give richer anthocyanins in its wines, which, in turn, give a more attractive color for consumers than *V. vinifera* wines [26].

### 3.2. Soluble Sugars, Organic Acids Profiles, and Related Gene Expressions

The soluble sugar and organic acid profiles of spine grapes are shown in Table 1. In grape berries, glucose and fructose are the main soluble sugars, and some table grape varieties do not contain any detectable sucrose [27]. The soluble sugar in grapes provides nutrients for the yeast, which can be converted into alcohols and esters by the yeast during the fermentation process, which affects the quality of the wine. The type and content of soluble sugar affect the fermentation process, which in turn affects the flavor quality of the wine [28]. The results of this study were consistent with previous research results, as only glucose and fructose were determined in the spine grapes. XZZ had the highest glucose and fructose content. G2 and G4 had the lowest fructose content. G4 and BY had the lowest glucose content. These results were consistent in that XZZ had the highest content of soluble solids, while G2 had the lowest. In general, the organic acids in grapes were mainly tartaric, malic, and citric acids, and these components play an important role in grape and wine quality [29]. The maturity and genotype of grapes affect the composition of these organic acids [29]. As shown in Table 1, malic acid was the main organic acid present in spine grapes and was detected in all varieties, while citric acid was only found in G2 and XZZ grapes. Yang et al. [24] also reported that the malic acid content was significantly higher than that of citric acid in Cabernet Sauvignon grapes. However, the malic acid content in the spine grapes ranged from 3.13 g/L to 4.22 g/L, which was significantly lower than in Cabernet Sauvignon grapes (11.53–13.57 g/L) [24]. Citric acid can also affect the acidity of wines and inhibit the activity of yeasts during fermentation, therefore, a higher content of citric acid in wine indicates adulteration [30]. The malic acid can be transferred to lactic acid by lactic acid bacteria, therefor, the concentration of malic acid decreases during fermentation. Tartaric acid is an organic acid unique to grapes and wine [16]. The tartaric acid is the main source of the acid taste of wine and plays an important role in maintaining the chemical stability of wine and its color or taste [30]. XZZ and G4 had the highest tartaric acid content, while the lowest tartaric acid content was detected in G2. These results indicate that there were differences in soluble sugar and organic acid composition between spine grape varieties and *Vitis vinifera* cultivars.

**Figure 1:**
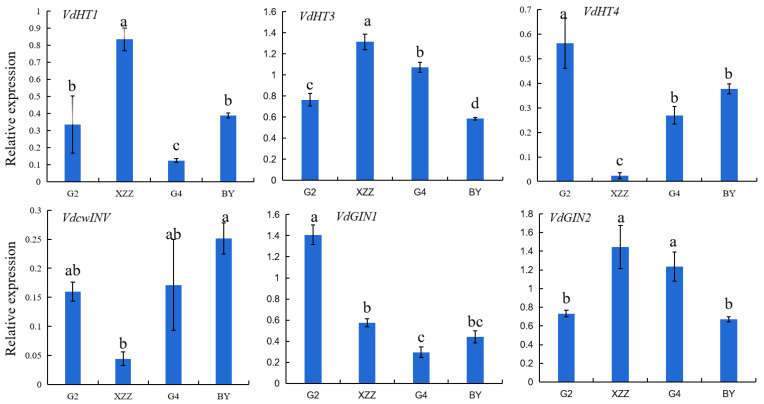
Expression levels of sugar unloading-associated genes in spine grapes. Values presented are means ± SD (*n* = 3). *VdGAPDH* was used as reference gene. Different letters indicate significant differences among four spine grapes using Duncan’s test (*p* < 0.05). G2: Gaoshan #2, G4: Gaoshan #4, XZZ: Xiangzhenzhu, BY: Baiyu.

The sugar in grape berries is transported in the form of sucrose from the source organs (leaves) through the phloem to the sieve element/companion cell (SE/CC) complex in the phloem of the fruit vascular bundle, and then unloaded from the sieve–companion cell complex and enters the sink cells [31]. The expressions of sugar unloading genes play a key regulatory role in the process of sugar transport. Thus, to further reveal the mechanism of the differences in varieties in soluble sugar profiles, the expression levels of six genes (*VdHT1*, *VdHT3*, *VdHT4*, *VdcwINV*, *VdGIN1*, and *VdGIN2*) involved in sugar unloading pathway were measured. As shown in Figure 1, the expression levels of *VdHT1*, *VdHT3*, and *VdGIN2* in XZZ grapes were significantly higher than those in other varieties, while the expression levels of *VdHT4* and *VdcwINV* in XZZ grapes were significantly lower than those in other varieties. *VdHT* genes are monosaccharide transporter proteins, and *VdcwINV*, *VdGIN1*, and *VdGIN2* are invertase genes [32]. The higher expression of *VdHT1* and *VdHT3* might increase the glucose content in grapes [24]. G2 grapes had the highest expression levels of *VdHT3* and *VdGIN1*, with the lowest levels of *VdHT1* and *VdGIN2*. The expression level of *VdcwIVN* was higher in BY grapes than in other varieties. Previous studies revealed that VvSK1, a sugar inducible protein kinase, may regulate the expressions of *VvHTs* and thus the accumulation of sugar in grape cells [33]. These results suggest that the accumulation mechanism of monosaccharides in different spine grape varieties might be regulated by other pathways. In grapes, the VvcwINV protein regulated the metabolism of sucrose, and VvGINs proteins were involved in the accumulation of hexose [34,35]. The expressions of *VdcwINV* and *VdGINs* might regulate the accumulation of sucrose in spine grapes, so sucrose was not detected. Similar results were also reported for Cabernet Sauvignon grapes [24].

### 3.3. Anthocyanin Profiles and Related Gene Expressions

The anthocyanin profiles of grapes may be affected by many factors, such as variety, management, temperature, and light [17]. In the present study, the anthocyanin profiles of four Chinese wild spine grape varieties were analyzed. The identification of anthocyanins was qualitatively determined by using the standard sample malvidin-3-glucoside, and then other anthocyanin components were identified according to references, which reported that the peak sequences of nine kinds of anthocyanins were: Delphinidin-3-glucoside (Dp), Cyanidin-3-glucoside (Cy), Petunidin-3-glucoside (Pt), Peonidin-3-glucoside (Pn), Malvidin-3-glucoside (Mv), Peonidin-3-acetly-glucoside (Pn-AG), Malvidin-3-acetly-glucoside (Mv-AG), Peonidin-3-coumayl-glucoside (Pn-CG) and Malvidin-3-coumayl-glucoside (Mv-CG) [36,37,38]. As the results in Table 2 show, a total of nine anthocyanin types were detected. There were no anthocyanins detected in the white spine grape (BY). Interestingly, cyanidin-3-glucoside and malvidin-3-acetyl-glucoside were the most abundant anthocyanins in the other three spine grapes, while malvidin-3-glucoside is the most abundant anthocyanin in *Vitis vinifera* grapes [17]. The Mv derivative content—including malvidin-3-glucoside, malvidin-3-acetyl-glucoside, and malvidin-3-coumayl-glucoside—was higher than any other kind of anthocyanin in spine grapes. The cyanidin-3-glucoside content in the spine grapes was second only to the content of malvidin-3-acetyl-glucoside. The three spine grapes had a few Cy, Dp, Pt, and Pn derivatives. Conversely, the content of Cy, Dp, Pt, and Pn derivatives in *Vitis vinifera* and *Vitis quinquangularis* cultivars is high, while the Mv derivative content is very low [4,25]. These results indicate that *Vitis davidii* cultivars might have different anthocyanin component characteristics than *Vitis vinifera* and *Vitis quinquangularis* varieties. More research is needed to reveal the anthocyanin properties of *Vitis davidii* cultivars. A recent study reported that some grape species native to China, including *V. davidii* and *V. amurensis*, had higher total anthocyanin contents (TAC) than *V. vinifera* grapes [4]. These indicated that spine wines might have similar health benefits since their anthocyanin contents were competitive with *V. vinifera* grape wines. In the present study, we also found that the anthocyanins contents in spine grapes were significantly higher than *V. vinifera* grapes, this might indicate that spine wines had richer anthocyanins and a more attractive color for consumers than *V. vinifera* wines [26].

**Figure 2:**
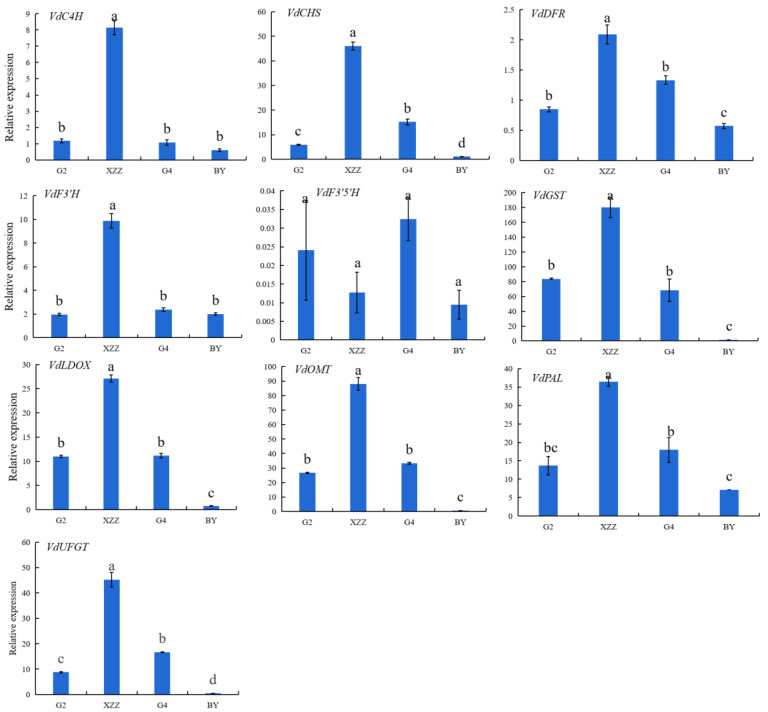
Expression levels of key genes involved in anthocyanin biosynthesis of spine grapes. Values presented are means ± SD (*n* = 3). *VdGAPDH* was used as reference gene. Different letters indicate significant differences among four spine grapes using Duncan’s test (*p* < 0.05). G2: Gaoshan #2, G4: Gaoshan #4, XZZ: Xiangzhenzhu, BY: Baiyu.

To better understand the mechanism of the varietal differences in anthocyanin profiles, the transcript levels of 10 key genes (*VdPAL*, *VdC4H*, *VdCHS*, *VdF3′H*, *VdF3′5′H*, *VdDFR*, *VdLDOX*, *VdUFGT*, *VdOMT*, and *VdGST*) involved in the anthocyanin biosynthesis pathway were analyzed. As Figure 2 shows, the expression levels of *VdPAL*, *VdC4H*, *VdCHS*, *VdF3′H*, *VdDFR*, *VdLDOX*, *VdUFGT*, *VdOMT*, and *VdGST* genes were highest in XZZ grapes, followed by G4 grapes. The transcript level of the *VdF3′5′H* gene was the lowest among the 10 genes in all spine grape varieties. Although anthocyanin biosynthesis genes were also expressed in white varieties (BY), these levels were very low. The product of *PAL*, *C4H*, and *CHS* acts upstream of the phenylalanine metabolic pathway, and its expressions were significantly different among grape varieties [39]. Our results also support this conclusion. The expression levels of *VdGST* and *VdOMT* were the highest among the 10 genes in all spine grapes, especially in XZZ grapes. These two genes play an important role in the biosynthesis of anthocyanins, so the high expression levels of *VdGST* and *VdOMT* may be the main reason for the high anthocyanin content of these grapes [18]. Transcriptomics revealed that the difference in gene expressions between varieties might be the direct cause of the differences in anthocyanin composition [29].

### 3.4. Glycosidically Bound Volatiles and Related Gene Expressions

The GBV profiles among the different spine grapes were determined, and the results are shown in Table 3. A total of 27 kinds of GBVs, including alcohols, monoterpenes, esters, aldehydes, ketones, and phenolic acid, were identified in spine grapes. These GBVs could be converted to their free-form volatiles by enzymatic hydrolysis, and the bound volatiles could contribute to the aromatic characteristics of grapes and wines, especially the monoterpenes, alcohols, and esters [40]. In the present study, BY had the highest monoterpene and alcohol content, followed by G4. The main monoterpenes in spine grapes were α-Terpineol, citronellol, terpinolene, and linalool, while benzyl alcohol was the most abundant alcohol; these bound volatiles could give the grapes and their wines a more floral and fruitier aroma [41]. BY and G4 had higher α-terpineol, citronellol, terpinolene, and benzyl alcohol contents than G2 and XZZ. Geraniol was only detected in BY and G4. For esters, methyl salicylate accounted for about 60%–80% of esters present, but no methyl salicylate was detected in XZZ grapes. BY had the highest methyl salicylate content, followed by G2. The ester content in XZZ grapes was the lowest among the four spine grape varieties. In addition, two ketone and phenolic acid-bound aromas were also detected in the tested grapes. No ketones were detected in BY, however, although mequinol was only detected in BY grapes. Overall, BY and G4 grapes had the highest bound volatiles content, while XZZ had the lowest. BY and G4 had rich bound volatiles, which might give the grapes more aromatic characteristics [14]. The GBVs in ripe grapes might relate to the concentration of free volatiles, and more research is needed to reveal the relationships and impact factors for the profiles of free and bound volatiles [15]. Compared with *V. vinifera* grapes, such as Cabernet Sauvignon, the spine grapes had a richer bound aroma, which might give the wine a richer aroma type, such as floral, fruity, etc.

**Figure 3:**
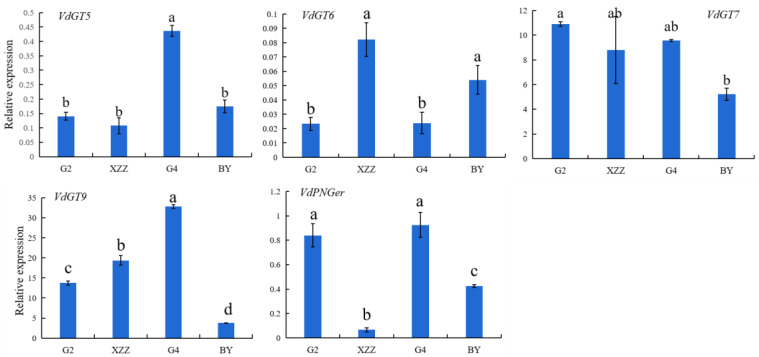
Expression levels of *VdGT5*, *VdGT6*, *VdGT7*, *VdGT9* and *VdPNGer*, which are associated with glycosidically bound volatiles biosynthesis of spine grapes. Values presented are means ± SD (*n* = 3). *VdGAPDH* was used as reference gene. Different letters indicate significant differences among four spine grapes using Duncan’s test (*p* < 0.05). G2: Gaoshan #2, G4: Gaoshan #4, XZZ: Xiangzhenzhu, BY: Baiyu.

To analyze the expression patterns of key genes involved in the GBV biosynthesis pathway in spine grapes, the transcript levels of five genes (*VdGT5*, *VdGT6*, *VdGT7*, *VdGT9*, and *VdPNGer*) were determined. As Figure 3 shows, the expression levels of *VdGT5*, *VdGT9*, and *VdPNGer* were higher in G4 grapes than in the other varieties. BY had the highest transcript level of *VdGT6* but lower expressions of *VdGT6*, *VdGT9*, and *VdPNGer*. XZZ had the highest expression level of *VdGT6* but the lowest expression levels of *VdGT5* and *VdGT9*. The expression levels of *VdGT9* and *VdGT7* were the highest among the five genes in all spine grapes, especially in G4 and G2 grapes, respectively. The GTs (monoterpenol *β*-d glucosyltransferases) proteins could catalyze the GBV biosynthesis in grapes [42]. Many studies have reported a positive correlation between the expression levels of *GTs* genes and the concentrations of GBVs [43,44]. The difference in *VdGTs* gene expressions between varieties might be the main reason for variation in GBVs between varieties [43,45].

### 3.5. Multivariate Statistical Analysis

**Figure 4:**
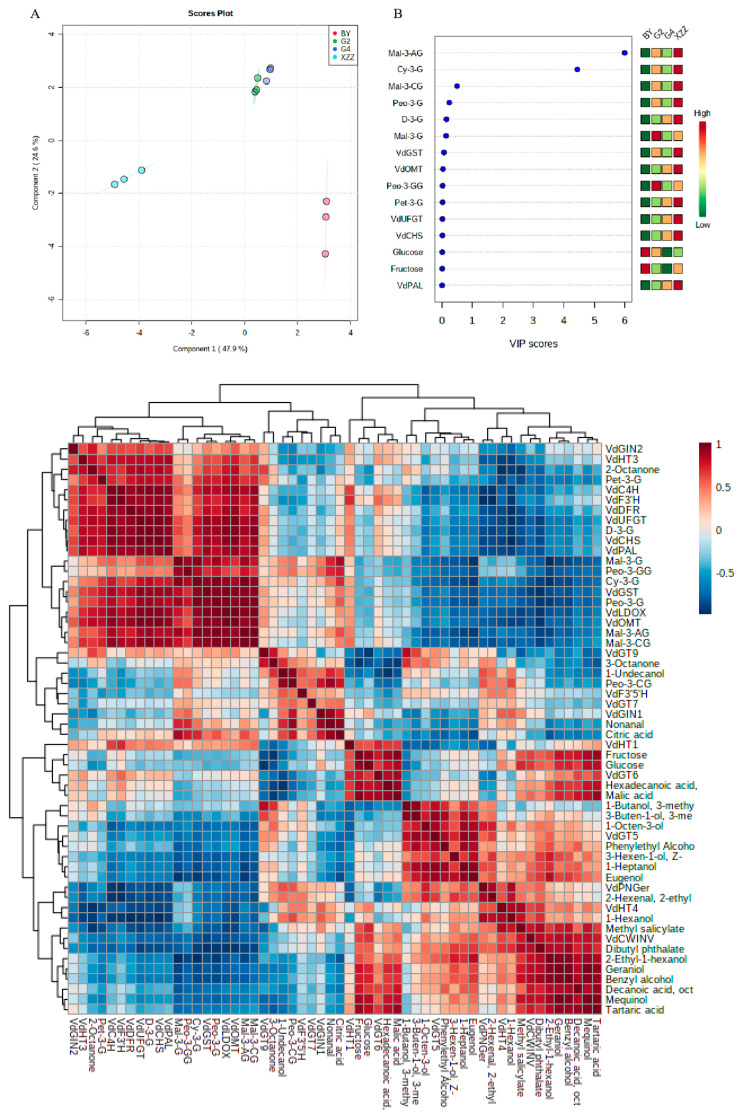
Multivariate statistical analysis. (**A**) Score plot of partial least-square discriminant analysis (PLS-DA) using metabolites and gene expression levels researched in this study. The PLS regression is performed using the plsr function provided by R pls package. The classification and cross-validation are performed using the corresponding wrapper function offered by the caret package provided by MetaboAnalyst. To assess the significance of class discrimination, a permutation test was performed. In each permutation, a PLS-DA model was built between the data (*X*) and the permuted class labels (*Y*) using the optimal number of components determined by cross validation for the model based on the original class assignment. In addition, test statistics for measuring the class discrimination were performed based on prediction accuracy during training. (**B**) Selected compounds based on VIP scores. Variable importance in projection (VIP) is a weighted sum of squares of the PLS loadings taking into account the amount of explained *Y*-variation in each dimension. VIP scores are calculated for each component. The colored boxes on the right indicate the relative concentration of the corresponding metabolites and gene expressions in each group under study. (**C**) Correlation analysis of metabolites and gene expression levels. G2: Gaoshan #2, G4: Gaoshan #4, XZZ: Xiangzhenzhu, BY: Baiyu.

To better understand the relationship between metabolites and gene expressions determined in the present study, multivariate statistical analysis was performed. As Figure 4 and Figure S2 (Supplementary Materials) show, the partial least-square discriminant analysis (PLS-DA) and correlation analysis were performed with the data concerning soluble sugars, organic acids, anthocyanins, GBVs, and related gene expressions. The first two components accounted for 72.5% of total variance (47.9% and 24.6%, respectively). PLS-DA analysis showed that the four spine grape varieties were distinguished from each other, which indicates that they possessed significant differences in metabolite and gene expression levels. The G2 and G4 groups were located on the positive side of the first and second components, and the distance was close, indicating that the metabolite and gene expression differences between the two varieties were not obvious; however, the BY and XZZ groups were located on the positive and negative sides of the first component, respectively, and were far away, indicating that there was a significant difference in metabolites and gene expressions between these two varieties.

To better analyze the differences in metabolites and gene expressions among the four varieties, 15 metabolites or genes with significant differences among the varieties were screened. Interestingly, the anthocyanin content and related gene expression levels were the most significant among the four varieties, especially malvidin-3-acetyl-glucoside and cyanidin-3-glucoside. These results further indicate that the characteristic anthocyanins in spine grapes were malvidin-3-acetyl-glucoside and cyanidin-3-glucoside, which is different from *Vitis vinifera* grapes [4,17]. The glucose and fructose content also contributed greatly to the distinction between varieties, and there were no bound volatiles in the screened components.

Correlation analysis revealed the relationship between metabolites and related gene expressions. The positive correlations between the expression level of *VdGT9* and bound volatiles contents, such as eugenol, 1-heptanol, 3-methyl-3-buten-1-ol, and 3-methyl-1-butanol, were detected. The expressions of *VdGT5* and *VdGT6* were significantly related to the concentrations of mequinol, benzyl alcohol, 1-hexanol, isogeraniol, citronellol, and linalool. Our results were consistent with those of Yue et al. [11], which reported that the expression levels of *GTs* genes were significantly related to the concentrations of GBVs. In addition, the concentrations of malvidin-3-acetyl-glucoside and cyanidin-3-glucoside were significantly related to the expressions of anthocyanin biosynthesis genes, especially *VdGST*, *VdF3′H*, *VdOMT*, *VdLDOX*, and *VdUFGT*. These genes play important roles in the biosynthesis and proportion of individual anthocyanins [46]. The difference in anthocyanin components in different spine grape varieties might therefore be caused by the difference in gene expression. We also found that sugar and organic acids were correlated with GBVs and anthocyanin contents. For example, there was a significant positive correlation between glucose and fructose content and mequinol, geraniol, benzyl alcohol, and 2-ethyl-1-hexanol. These results indicate that there may be a close relationship between primary and secondary metabolites [24].

## 4. Conclusions

In conclusion, targeted metabolomics and transcription level analysis revealed that there were significant differences in the soluble sugars, organic acids, anthocyanins, GBVs, and gene expressions among the four spine grape clones. The XZZ grape had the highest soluble sugar and organic acid content. The characteristic anthocyanins in spine grapes were malvidin-3-acetyl-glucoside and cyanidin-3-glucoside, and the XZZ variety had the highest individual anthocyanin content. The rich anthocyanins might give spine wines richer anthocyanins and a more attractive color for consumers than *V. vinifera* wines. Spine grapes are rich in GBVs, which give wines more aroma type, such as floral, fruity, etc. G4 and BY grapes had the highest GBV concentrations. Multivariate statistical analysis revealed a close relationship between metabolites and gene expression in spine grapes. The results of this study help remedy the lack of knowledge about wild spine grape resources. Similar studies will ultimately benefit the promotion of spine grapes, and our data might be useful for the development and use of wild grape resources. In addition, considering rich anthocyanin and GBV contents, spine grapes might have potential for winemaking.

## Figures and Tables

**Table 1:** Soluble sugar and organic acid composition of Chinese wild spine grapes (g/L).

Cultivars	Fructose	Glucose	Tartaric Acid	Malic Acid	Citric Acid
G2	64.31 ± 2.04 ^c^	71.03 ± 3.41 ^ab^	1.21 ± 0.08 ^bc^	3.13 ± 0.22 ^bc^	0.11 ± 0.02 ^a^
XZZ	80.73 ± 4.18 ^a^	75.89 ± 8.51 ^a^	1.43 ± 0.02 ^a^	4.22 ± 0.20 ^a^	0.06 ± 0.01 ^b^
G4	65.42 ± 0.61 ^c^	63.40 ± 0.02 ^c^	1.42 ± 0.08 ^a^	3.35 ± 0.27 ^b^	-
BY	68.33 ± 1.81 ^b^	63.59 ± 1.98 ^c^	1.33 ± 0.00 ^b^	3.17 ± 0.12 ^bc^	-

Note: All values shown are mean ± SD, *n* = 3. Lowercase letters indicate significant (*p* < 0.05); “-” means not detected; G2: Gaoshan #2; G4: Gaoshan #4; XZZ: Xiangzhenzhu; BY: Baiyu.

**Table 2:** Anthocyanin profiles of Chinese wild spine grapes (mg/L).

Anthocyanins	G2	XZZ	G4	BY
Delphinidin-3-glucoside	21.20 ± 7.64 ^c^	122.91 ± 0.97 ^a^	88.42 ± 3.19 ^b^	nd
Cyanidin-3-glucoside	2764.26 ± 2.13 ^c^	4232.11 ± 7.79 ^a^	3935.36 ± 0.29 ^b^	nd
Petunidin-3-glucoside	8.22 ± 11.63 ^b^	19.08 ± 0.36 ^b^	35.84 ± 0.17 ^a^	nd
Peonidin-3-glucoside	114.69 ± 1.90 ^c^	211.47 ± 1.43 ^b^	242.04 ± 6.99 ^a^	nd
Malvidin-3-glucoside	144.05 ± 0.57 ^c^	151.29 ± 1.11 ^b^	159.65 ± 0.04 ^a^	nd
Peonidin-3-acetly-glucoside	31.38 ± 20.81 ^a^	28.48 ± 0.32 ^a^	29.21 ± 0.02 ^a^	nd
Malvidin-3-acetly-glucoside	2807.84 ± 13.63 ^c^	5571.95 ± 2.90 ^a^	3015.96 ± 1.36 ^b^	nd
Peonidin-3-coumayl-glucoside	15.17 ± 0.07 ^a^	10.12 ± 0.12 ^c^	14.57 ± 0.01 ^b^	nd
Malvidin-3-coumayl-glucoside	156.44 ± 1.56 ^c^	435.45 ± 0.04 ^a^	226.41 ± 0.01 ^b^	nd

Note: All values shown are mean ± SD, *n* = 3; Lowercase letters indicate significant (*p* < 0.05); nd means not detected; G2: Gaoshan #2; G4: Gaoshan #4; XZZ: Xiangzhenzhu; BY: Baiyu.

**Table 3:** Glycosidically bound volatiles of Chinese wild spine grapes (μg/L).

Volatiles	G2	XZZ	G4	BY
3-methyl-1-Butanol	-	-	2.16 ± 0.10 ^b^	-
3-methyl-3-Buten-1-ol	-	-	3.34 ± 0.28 ^a^	0.63 ± 0.06 ^b^
1-Octen-3-ol	1.60 ± 0.01 ^b^	1.12 ± 0.56 ^b^	2.61 ± 0.75 ^a^	1.25 ± 0.12 ^b^
1-Heptanol	1.35 ± 0.01 ^b^	1.26 ± 0.09 ^b^	2.46 ± 0.23 ^a^	1.31 ± 0.04 ^b^
Geraniol	-	-	0.24 ± 0.04 ^b^	0.91 ± 0.13 ^b^
Linalool	1.32 ± 0.01 ^b^	1.20 ± 0.02 ^b^	1.21 ± 0.03 ^b^	2.51 ± 0.01 ^a^
α-terpineol	1.01 ± 0.02 ^b^	1.21 ± 0.01 ^b^	1.32 ± 0.04 ^b^	3.21 ± 0.04 ^a^
Citronellol	2.31 ± 0.01 ^c^	2.51 ± 0.01 ^c^	3.01 ± 0.01 ^b^	4.31 ± 0.01 ^a^
Isogeraniol	1.31 ± 0.01 ^b^	0.92 ± 0.01 ^b^	2.14 ± 0.01 ^a^	2.35 ± 0.01 ^a^
Terpinolene	2.31 ± 0.11 ^c^	3.21 ± 0.01 ^b^	3.65 ± 0.20 ^ab^	4.31 ± 0.22 ^a^
Benzyl alcohol	4.92 ± 0.01 ^c^	4.20 ± 1.43 ^c^	7.17 ± 1.16 ^c^	11.82 ± 1.50 ^b^
Phenylethyl alcohol	0.49 ± 0.01 ^b^	0.66 ± 0.26 ^b^	1.32 ± 0.20 ^b^	0.69 ± 0.02 ^b^
1-Hexanol	5.36 ± 0.08 ^a^	1.46 ± 0.61 ^d^	4.14 ± 0.26 ^b^	3.08 ± 0.38 ^c^
2-Ethyl-1-hexanol	-	-	4.27 ± 0.45 ^c^	5.92 ± 1.09 ^a^
(Z)-3-Hexen-1-ol	0.43 ± 0.09 ^bc^	0.17 ± 0.02 ^bc^	1.15 ± 0.14 ^a^	0.62 ± 0.54 ^ab^
**Total alcohols and monoterpene**	22.41	17.92	40.19	42.92
1-Undecanol	7.45 ± 0.10 ^a^	1.05 ± 0.25 ^d^	5.70 ± 1.21 ^b^	-
Nonanal	2.22 ± 0.17 ^b^	0.51 ± 0.18 ^c^	-	-
2-ethyl-2-Hexenal	4.12 ± 1.12 ^d^	-	5.25 ± 0.36 ^c^	2.15 ± 1.22 ^e^
**Total aldehyde**	13.79	1.56	10.95	2.15
Hexadecanoic acid, ethyl ester	-	1.26 ± 0.09 ^b^	-	1.20 ± 0.20 ^b^
Methyl salicylate	9.54 ± 0.59 ^a^	-	3.44 ± 0.15 ^c^	10.00 ± 2.41 ^a^
Dibutyl phthalate	2.15 ± 0.09 ^a^	1.62 ± 0.38 ^c^	2.65 ± 0.23 ^a^	2.16 ± 0.25 ^a^
Decanoic acid, octyl ester	-	0.54 ± 0.31 ^bc^	0.88 ± 0.06 ^bc^	1.78 ± 0.62 ^a^
**Total ester**	11.69	3.42	6.97	15.14
3-Octanone	2.28 ± 0.26 ^bc^	1.61 ± 0.93 ^c^	3.35 ± 1.20 ^ab^	-
2-Octanone	-	10.19 ± 2.49 ^a^	7.12 ± 1.98 ^a^	-
**Total ketone**	2.28	11.8	10.47	-
Mequinol	-	-	-	8.17 ± 0.98 ^b^
Eugenol	1.85 ± 0.01 ^b^	1.85 ± 0.59 ^b^	4.65 ± 0.84 ^a^	2.67 ± 0.47 ^b^
**Total phenolic acid**	1.85	1.85	4.65	10.84
**Total GBVs**	52.02	36.55	73.95	71.05

Note: All values shown are mean ± SD, *n* = 3. Lowercase letters indicate significant (*p* < 0.05); “-” means not detected; GBVs: glycosidically bound volatiles; G2: Gaoshan #2; G4: Gaoshan #4; XZZ: Xiangzhenzhu; BY: Baiyu.

## Supplementary Materials

The following are available online at https://www.mdpi.com/2304-8158/9/10/1387/s1, Figure S1: Physicochemical parameters of Chinese wild spine grapes; Figure S2. The loadings plot of PLS-DA; Table S1: The primers used in this study.
