# Supplementary material for: Targeted Metabolomic and Transcript Level Analysis Reveals Quality Characteristic of Chinese Wild Grapes (Vitis davidii Foex)
Source: Foods. 2020 Oct 1;9(10):1387. doi: 10.3390/foods9101387 (PMC7600675; doi:10.3390/foods9101387)
Supplement: Supplementary file 1 [file foods-09-01387-s001.pdf]

Supplementary Materials

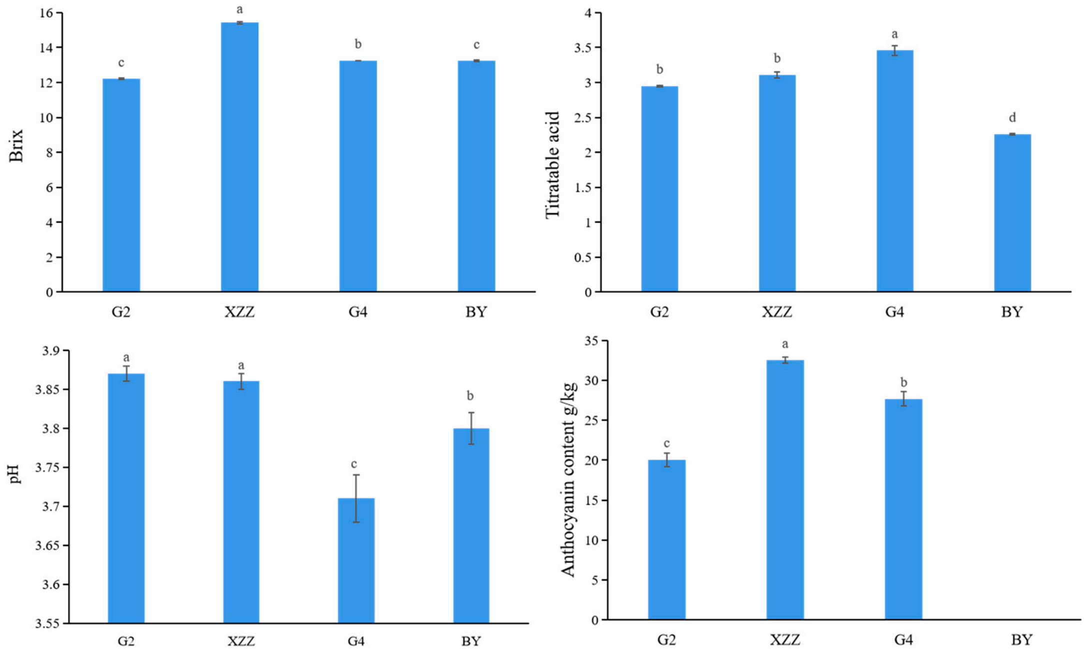

Figure S1. Physicochemical parameters of Chinese wild spine grapes.

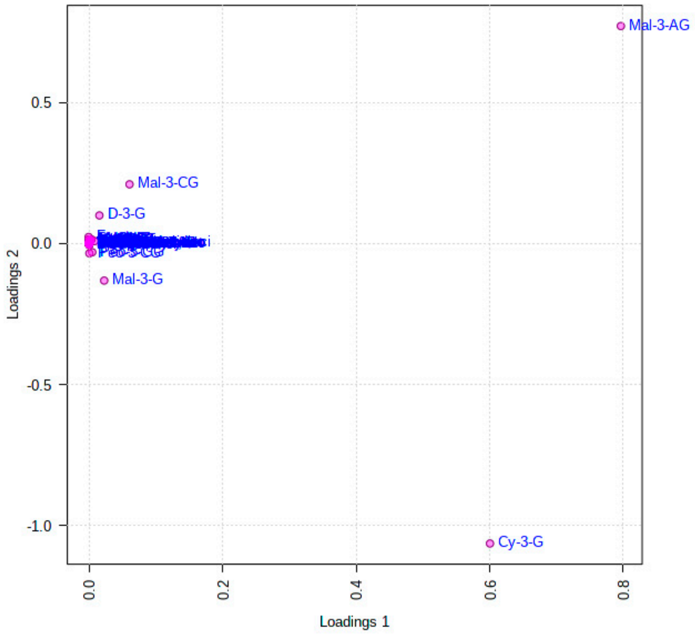

Figure S2. The loadings plot of PLS-DA.

Table S1. The primers used in this study.

**Primer**		**Primer sequence (From 5′ to 3′)**
VdPNGer	F	ATCTTCCTTTGTCGCTCCTT
	R	CCGCATGTGGAGATAGAGTT
VdGT5	F	GCTCCATCTCCTCTGCTCAAA
	R	GAAAGCACAAGGTCCTCT
VdGT6	F	GGTTCCCTGGTTGGCAATTT
	R	GCACCCGCCCCACAACCTT
VdGT7	F	TTTAGCACCACCGGAACCGGA
	R	TTCAAGCACTGAGTTCCACCCA
VdGT9	F	CCAAGTCACCGACGCCAAGTACC
	R	ACTCATCCTCCTGACCTCGTCCAC
VdHT1	F	GTCTATGTTTCAGGGTTTG
	R	AAGAAGATTTGGGCTATG
VdHT2	F	GTTGCCGTCAACTTCGCAAC
	R	GTTGCCGTCAACTTCGCAAC
VdHT3	F	AGAGGAACTATGGAGGTGG
	R	AACAAGGCAAGCAACGAC
VdHT4	F	CTGATGTTGCAGCGTGTTC
	R	GGAGGCCATACCAACTACG
VdcwINV	F	ATGAATCATCTAGYGTGGAGCAC
	R	CTTAAACGATATCTCCACATCTGC
VdGIN1	F	CCATCTCCATCCCATCGTAACC
	R	GGCTATCCAAGTTTCCAACCAACC
VdGIN2	F	GAGCACAGTTCCAGTAATCAAAGG
	R	GTGAGGCGTAGTTTTAGGACTCC
VdPAL	F	CAACCAAGATGTGAACTCCTT

	R	TTCTCCTCCAAATGCCTC
VdC4H	F	GGCAAGCACAAAGAGCACAGAT
	R	TTCTTCTGGATGTGAGGGTGGTT
VdCHS	F	GTCTGAAGGAAGAGAAACTGAGAG
	R	CCAGGATAAACAACACGCAT
VdF3′H	F	CAACAAGAGCTGGACGCAGT
	R	AGCCGTTGATCTCACAGCTC
VdF3′5′H	F	AAACCGCTCAGACCAAAACC
	R	ACTAAGCCACAGGAAACTAA
VdDFR	F	GGCCAAATCAAACTACCAGA
	R	GAAACCTGTAGATGGCAGGA
Primer		Primer sequence (From 5′ to 3′)
VdLDOX	F	AGGGAAGGGAAAACAAGTAG
	R	ACTCTTTGGGGATTGACTGG
VdUFGT	F	GGGATGGTAATGGCTGTGG
	R	ACATGGGTGGAGAGTGAGTT
VdOMT	F	GAGAGCAGGCAGAGTCCATC
	R	CACCATAAGCAAACCCTAAACC
VdGST	F	GAGGAGAAAGCGGTAGTTG
	R	CCCATTCGAGGTAGGATC
VdGAPDH	F	TTCTCGTTGAGGGCTATTCCA
	R	CCACAGACTTCATCGGTGACA
